# A Great Delight

**Published:** 1873-02

**Authors:** 


### A GREAT DELIGHT

Of a cold winter's morning is to dress by a good blazing fire. There is no possible advantage in getting out of a warm bed and washing and dressing in the cold; to do so is a positive danger to the old, the young, the feeble, and the sedentary. There is a parcel of numskulls over the land who seem to take a delight in finding out what is comfortable, and then proclaiming its unhealthfulness, as the genius, whoever he may be, has proclaimed himself a born ass by stating that tomatoes are pernicious as food, because in a single observed case or two, they seemed to have had an ill effect in consequence of the person having eaten a peck of them at a meal.

Another fellow says that if people don't eat more grapes, and writes a book to prove it, that their bones will become so brittle, they will break into pieces every time they stub their toes. Poor fellow, he died of the "pure juice of the grape" before he reached three score. One thing is certain: everything that is "good," has been given us "richly to enjoy."

